# Supplementary figures and images for: Using null models to infer microbial co-occurrence networks
Source: PLoS One. 2017 May 11;12(5):e0176751. doi: 10.1371/journal.pone.0176751 (PMC5426617; doi:10.1371/journal.pone.0176751)

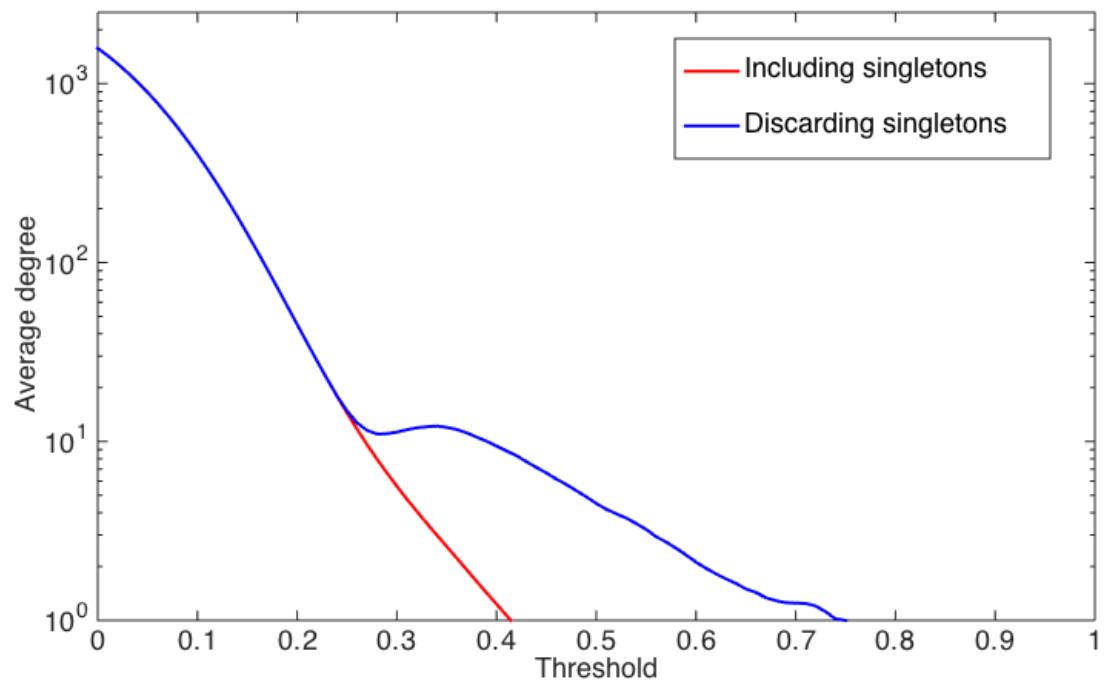

Supplement: S1 Fig — When singleton nodes (nodes of degree zero) are maintained in the network, we see the pattern in the red line, as nodes with degree zero contribute to lowering the average degree. When singleton nodes are discarded, we see the blue line where the average degree is inflated. In the main text, the blue line, with singleton nodes removed, is reported. (PDF) [file pone.0176751.s001.pdf]

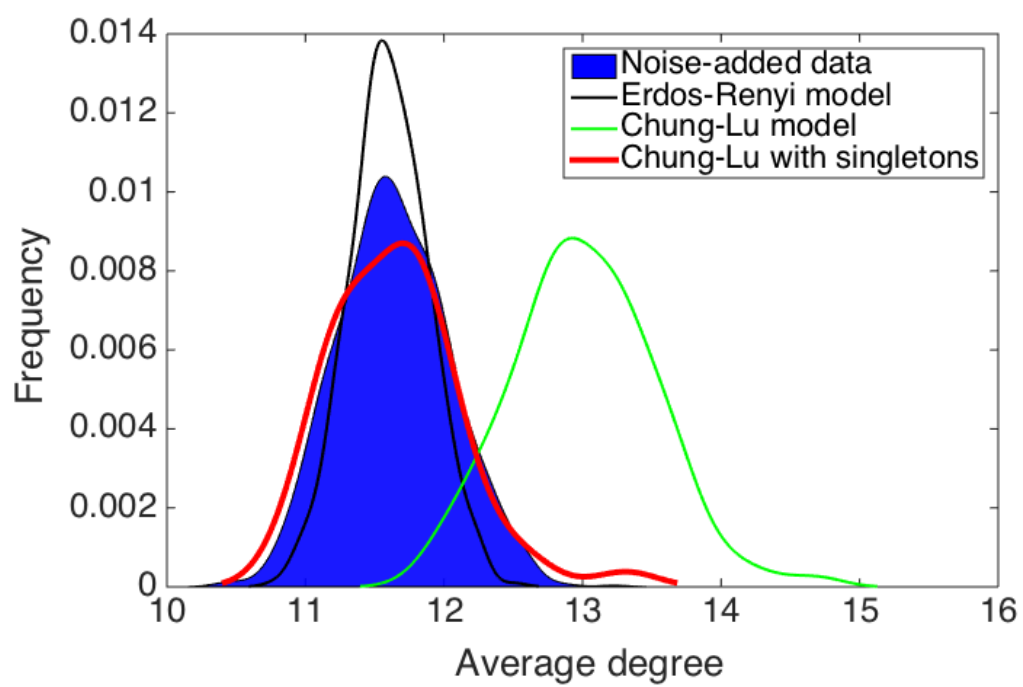

Supplement: S2 Fig — This figure shows the Chung-Lu random graph has an artificially inflated average degree. This is due to the removal of singletons from the graph (red line includes singletons in the degree calculation; green line excludes them) (PDF) [file pone.0176751.s002.pdf]
